# Predictive value of valvular calcification for the recurrence of persistent atrial fibrillation after radiofrequency catheter ablation

**DOI:** 10.1002/clc.24176

**Published:** 2023-11-07

**Authors:** Tong Liu, Meng‐Meng Li, De‐Yong Long, Jie Yang, Xin Zhao, Chang‐Yi Li, Wei Wang, Chen‐Xi Jiang, Ri‐Bo Tang

**Affiliations:** ^1^ Department of Cardiology, Beijing Institute of Heart, Lung and Blood Vessel Diseases, Beijing Anzhen Hospital Capital Medical University Beijing China

**Keywords:** persistent atrial fibrillation, radiofrequency catheter ablation, recurrence, score system, valvular calcification

## Abstract

**Background:**

Valvular calcification (VC) is an independent risk factor for cardiovascular diseases. The relationship between VC and atrial fibrillation is not clear.

**Hypothesis:**

We treated the aortic valve, mitral valve, and tricuspid valve as a whole and considered the possible association between VC and recurrence of persistent atrial fibrillation (PsAF) after radiofrequency catheter ablation (RFCA).

**Methods:**

This study involved 2687 PsAF patients who underwent RFCA. Data were collected to explore the relationship between VC and outcome. VC was defined by echocardiography in aortic valve, mitral valve, or tricuspid valve. After 1 year follow‐up, subgroup analysis, mixed model regression analysis, and score system analysis were performed. The external validation of 133 patients demonstrated the accuracy of this clinical prediction model.

**Results:**

Overall, 2687 inpatients were assigned to the recurrence group (*n* = 682) or the no recurrence group (*n* = 2005) with or without VC. Compared to patients with no recurrence, the incidence of VC was higher in recurrence patients. Recurrence was present in 18.5%, 34.9%, 39.3%, and 52.0% of the four groups, which met VC numbers of 0, 1, 2, and 3, respectively. After adjustment for potential confounding factors, VC was an independent risk factor for AF recurrence in several models. For multivariable logistic regression, a scoring system was established based on the regression coefficient. The receiver operating characteristic area of the scoring system was 0.787 in the external validation cohort.

**Conclusions:**

VC was an independent risk factor for AF recurrence in PsAF after RFCA. The scoring system may be a useful clinical tool to assess AF recurrence.

## Graphical abstract

In 2687 persistent atrial fibrillation (PsAF) undergoing radiofrequency catheter ablation, valvular calcification was an independent risk factor for AF recurrence after 1‐year follow‐up. For multivariable logistic regression, a scoring system was established based on the regression coefficient.

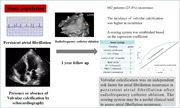

## 1 INTRODUCTION

As a common arrhythmia, atrial fibrillation (AF) is typified by a heart rate (HR) that is irregular and typically rapid. Over the past decade, the heightened occurrence of AF has been an important public health challenge.^1^ Radiofrequency catheter ablation (RFCA) has become an important treatment for AF, especially in heart failure (HF).^2^ As a type of AF, persistent AF (PsAF) was defined as AF lasting more than 7 days. Due to the complex pathogenesis, myocardial fibrosis and disorder of electrical activity, it is also challenging to maintain sinus rhythm after RFCA in PsAF.^3^

Valvular calcification (VC) is characterized by calcium deposition, osteogenic lesions, and fibrocalcific changes in the valve leaflet, which are also tightly associated with cardiovascular diseases.^4^ Fashanu et al. revealed that VC was associated with the incidence of HF and left ventricular function.^5^ A cohort study found that aortic valve calcification (AVC) showed predictive value for the incidence of major adverse cardiovascular events.^6^ Patients with valvular diseases were associated with the incidence of AF. In addition, VC was also an independent predictor of the incidence of AF. In the Framingham study, there were 42 individuals with incident AF, and the hazard ratio was 1.6 after an analysis.^7^

The RFCA of PsAF was also a challenge. Nevertheless, the relationship between the outcome of PsAF RFCA and VC is still unknown. We investigated the association of VC with the recurrence of PsAF after RFCA. In addition, we explored the influencing factors and established a scoring system to identify high‐risk patients.

## 2 PATIENTS AND METHODS

### 2.1 Study cohort

A total of 2687 inpatients with PsAF who had undergone RFCA were consecutively enrolled in this study from January 2018 to 2022 at The Anzhen Hospital. In addition, as external validation, 133 patients were enrolled between February 2022 and May 2022, and the predictive value of the scoring system was explored. According to the VC by echocardiography, the patients were assigned to the calcification group (*n* = 327) or noncalcification group (*n* = 2360). PsAF was defined as a period of rapid, irregular atrial rhythm lasting at least 7 consecutive days.^8^ VC was defined as the presence of bright echoes on the aortic valve, mitral valve, or tricuspid valve.^9^ The major exclusion criteria included the following: paroxysmal AF, history of AF ablation, rheumatic heart disease, severe stenosis or regurgitation of the heart valve, and severe disease in other systems. The detailed recruitment process is shown in Figure S1. The study protocol was approved by the Institutional Review Board of The Anzhen Hospital, Beijing, China.

### 2.2 Data collection

Data regarding demographic characteristics, clinical features, and laboratory examinations were collected for all subjects, such as sex, age, past medical history, smoking status, body mass index (BMI), blood pressure, HR, systolic blood pressure (SBP), left ventricular ejection fraction (LVEF), and albumin. The diagnostic criteria of the classical risk factors, including coronary artery disease (CAD),^10^ HF,^11^ stroke,^12^ and diabetes mellitus (DM),^13^ were followed according to the authoritative international guidelines.

### 2.3 RFCA procedure

Before RFCA, oral anticoagulation was given for at least 4 weeks. Transesophageal echocardiography or intracardiac echocardiography was performed to exclude left atrial thrombus. During RFCA, heparin was administered to maintain an activated clotting time of more than 300 s. After RFCA, antiarrhythmic drugs and oral anticoagulation were given for at least 3 months. Under the CARTO mapping system (CARTO; Biosense Webster Inc.), RFCA was performed at a maximum temperature of 45°C, maximum power of 50 W, and flow rate of 15 mL/min, and included circumferential pulmonary vein isolation, linear ablations, or complex fractionated atrial electrograms. The end point was defined as the isolation of the pulmonary vein and restoration of sinus rhythm.

### 2.4 Follow‐up

Follow‐up was performed by telephone and outpatient clinics. Twenty‐four‐hour Holter monitoring was performed at 1, 2, 3, 6, and 12 months after discharge. In addition, if patients felt serious symptoms of arrhythmia, they were also required to undergo an electrocardiogram at the closest hospital. AF recurrence was defined as documented atrial tachycardia, atrial flutter, or AF for at least 30 s during the 1‐year follow‐up. The final diagnosis of recurrence was reviewed by two cardiologists.

### 2.5 Statistical analyses

The statistical computations were performed using SPSS software, version 26.0 (IBM Corp.). Continuous variables are reported as the means ± standard deviations for normally distributed data or medians and quartiles (quartile 1; quartile 3) for nonnormally distributed data and were compared using Student's *t*‐test for normally distributed data or Mann−Whitney *U* test for nonnormally distributed data; the Kolmogorov−Smirnov test was used to check normality. Discrete variables are expressed as frequencies and percentages and were compared using the *χ* ^2^ test. Multivariable logistic regression analyses were performed to detect the relationship between AF recurrence and VC after adjusting for potential confounding variables. There were seven mixed‐model regressions, which were adjusted for age, sex, ascending aorta inner diameter, left atrial diameter, LVEF, course of AF, BMI, HT, HF, CAD, DM, stroke, history of alcohol intake, SBP, DBP, HR, white blood cell, red blood cell, platelet, hemoglobin, lymphocytes, neutrophils, monocytes, alanine transaminase, aspartate aminotransferase, triglycerides, total cholesterol, high‐density lipoprotein, low‐density lipoprotein, urea, creatinine, uric acid, blood glucose, Na, K, total protein, albumin, CPVA, Roofline, mitral isthmus line, CTI, CFAE, superior vane cava, and others. These predictors of AF recurrence were assigned corresponding points based on their regression coefficient and produced a score system. Receiver operating characteristic (ROC) curves were constructed, and the areas under the curves (AUCs) were calculated to assess the discriminatory power of the scoring system. A two‐sided *p* value < 0.05 was considered statistically significant.

## 3 RESULTS

### 3.1 Baseline characteristics

According to the 1‐year follow‐up, 2687 patients were classified into a recurrence group (*n* = 682) and a no recurrence group (*n* = 2005). The ages were 61.3 ± 10.3 and 60.8 ± 10.3, respectively. For VC, there were 122 patients (21.8%) in the recurrence group and 205 patients (9.6%) in the no recurrence group (*p* < 0.001). Compared to no recurrence, the BMI, course of AF and LA were higher in recurrence for all patients (all *p* < 0.05). The incidence of DM and alcohol consumption were significantly higher in recurrence for all patients (all *p* < 0.05). The FBG, TG, TC, and LDL‐C levels were more elevated in patients with recurrence for all patients (all *p* < 0.001). In subgroup analysis, BMI, course of AF and LA were higher in recurrence patients for the VC subgroup and non‐VC calcification subgroup (all *p* < 0.001). Similarly, the recurrence group showed significantly higher FBG, TG, and TC levels than the no recurrence group for the calcification subgroup (all *p* < 0.05). Furthermore, there were no significant differences in ablation procedures in the overall group and subgroups (Table 1).

**Table 1:** Clinical characteristics in valvular calcification and no valvular calcification patients with recurrence and no recurrence.

Variables	Overall			Calcification			No calcification		
	Recurrence	No recurrence	*p* Value	Recurrence	No recurrence	*p* Value	Recurrence	No recurrence	*p* Value
	*n* = 682	*n* = 2005		*n* = 140	*n* = 187		*n* = 542	*n* = 1818	
Male, *n* (%)	482 (70.7)	1436 (71.6)	0.637	102 (72.9)	137 (73.3)	0.935	380 (70.1)	1299 (71.5)	0.545
Age (years)	61.3 ± 10.3	60.8 ± 10.3	0.317	64.5 ± 10.6	61.9 ± 9.3	0.018	60.5 ± 10.1	60.7 ± 10.4	0.620
BMI (Kg/m)^2^	25.9 ± 3.3	25.1 ± 2.6	<0.001	26.6 ± 3.3	24.9 ± 2.1	<0.001	25.7 ± 3.3	25.1 ± 2.6	<0.001
HT, *n* (%)	310 (45.5)	897 (44.7)	0.745	63 (45.0)	81 (43.3)	0.761	247 (45.6)	816 (44.9)	0.778
valvular calcification, *n* (%)	122 (21.8)	205 (9.6)	<0.001	‐	‐	‐	‐	‐	‐
Course of AF, *n* (%)	21.7 ± 6.8	18.3 ± 4.0	<0.001	21.9 ± 6.6	18.0 ± 4.6	<0.001	21.6 ± 6.9	18.4 ± 4.0	<0.001
Ascending aorta inner diameter (mm)	36.0 ± 2.9	35.8 ± 2.8	0.171	36.0 ± 3.0	36.0 ± 2.9	0.877	36.0 ± 2.9	35.8 ± 2.8	0.190
LA (mm)	39.8 ± 5.0	37.2 ± 5.0	<0.001	40.2 ± 5.0	37.4 ± 5.2	<0.001	39.6 ± 5.0	37.2 ± 5.0	<0.001
HF, *n* (%)	40 (5.9)	115 (5.7)	0.900	7 (5.0)	14 (7.5)	0.364	33 (6.1)	101 (5.6)	0.638
CAD, *n* (%)	108 (15.8)	314 (15.7)	0.914	25 (17.9)	31 (16.6)	0.761	83 (15.3)	283 (15.6)	0.886
DM, *n* (%)	151 (22.1)	369 (18.4)	0.033	48 (34.4)	35 (18.1)	<0.001	103 (19.0)	334 (18.4)	0.740
Stroke, *n* (%)	87 (12.8)	223 (11.1)	0.248	21 (15.0)	21 (11.2)	0.313	66 (12.2)	202 (11.1)	0.492
Alcohol consumption, *n* (%)	179 (26.2)	305 (15.2)	<0.001	42 (29.3)	32 (17.1)	0.009	138 (25.5)	273 (15.0)	<0.001
SBP (mmHg)	126.0 ± 15.9	126.7 ± 15.2	0.316	125.1 ± 17.7	127.3 ± 14.5	0.226	126.3 ± 15.4	126.7 ± 15.2	0.604
DBP (mmHg)	79.9 ± 12.1	80.7 ± 21.8	0.373	79.0 ± 12.9	79.4 ± 10.8	0.763	80.1 ± 11.9	80.8 ± 22.6	0.494
HR (bpm)	79.1 ± 14.3	79.0 ± 13.9	0.877	78.5 ± 14.7	78.6 ± 12.2	0.931	79.2 ± 14.2	79.0 ± 14.0	0.754
FBG (mmol/L)	6.43 ± 1.20	6.19 ± 1.05	<0.001	7.09 ± 1.23	6.37 ± 1.22	<0.001	6.26 ± 1.14	6.17 ± 1.03	0.077
TG (mmol/L)	1.53 ± 0.50	1.48 ± 0.46	0.011	1.76 ± 0.86	1.47 ± 0.28	<0.001	1.47 ± 0.34	1.48 ± 0.48	0.796
TC (mmol/L)	4.00 ± 1.05	3.86 ± 1.17	0.004	4.22 ± 0.84	3.89 ± 1.21	0.007	3.95 ± 1.09	3.86 ± 1.16	0.096
LDL‐C (mmol/L)	3.11 ± 1.08	2.93 ± 1.09	0.008	3.13 ± 1.03	3.07 ± 1.18	0.603	3.05 ± 1.09	2.92 ± 1.08	0.019
HDL‐C (mmol/L)	1.64 ± 0.39	1.63 ± 0.37	0.611	1.64 ± 0.35	1.61 ± 0.37	0.444	1.64 ± 0.38	1.64 ± 0.37	0.718
WBC (10^12^/L)	8.32 ± 2.03	8.27 ± 2.04	0.636	8.34 ± 2.13	8.35 ± 2.19	0.988	8.31 ± 2.00	8.27 ± 2.03	0.659
HGB (g/L)	143.0 ± 18.3	143.6 ± 18.0	0.825	142.1 ± 17.8	140.8 ± 20.5	0.563	143.3 ± 18.5	143.9 ± 17.7	0.509
PLT (10^9^/L)	175.3 ± 33.8	174.7 ± 33.7	0.704	172.6 ± 35.1	174.1 ± 31.9	0.671	176.0 ± 33.4	174.8 ± 33.8	0.461
RBC (10^12^/L)	4.19 ± 1.13	4.22 ± 1.13	0.463	4.24 ± 0.99	4.09 ± 1.04	0.201	4.15 ± 1.17	4.24 ± 1.14	0.257
Lymphocytes (10^12^/L)	2.11 ± 0.61	2.08 ± 0.58	0.414	2.00 ± 0.64	2.07 ± 0.55	0.327	2.13 ± 0.60	2.09 ± 0.58	0.106
Neutrophil (10^12^/L)	4.37 ± 1.54	4.38 ± 1.45	0.882	4.51 ± 1.73	4.25 ± 1.36	0.128	4.34 ± 1.49	4.40 ± 1.46	0.410
Monocytes (10^12^/L)	0.52 ± 0.14	0.51 ± 0.15	0.615	0.52 ± 0.15	0.53 ± 0.15	0.578	0.52 ± 0.14	0.51 ± 0.14	0.598
ALT (μmol/L)	23.0 ± 6.9	23.2 ± 7.2	0.535	22.7 ± 5.3	23.3 ± 5.5	0.322	23.1 ± 7.2	23.2 ± 7.4	0.763
LVEF (%)	54.9 ± 4.0	55.0 ± 3.9	0.567	54.8 ± 4.4	55.2 ± 3.9	0.456	54.9 ± 3.8	55.0 ± 3.9	0.722
AST (μmol/L)	15.2 ± 6.4	15.3 ± 5.6	0.813	15.4 ± 6.1	15.0 ± 4.2	0.474	15.2 ± 6.5	15.3 ± 5.8	0.639
Urea (mmol/L)	5.31 ± 1.27	5.35 ± 1.48	0.851	5.34 ± 1.23	5.40 ± 1.47	0.695	5.31 ± 1.29	5.34 ± 1.48	0.582
Cr (μmoI/L)	74.2 ± 12.2	74.8 ± 21.7	0.481	74.5 ± 12.0	74.5 ± 13.4	0.968	74.0 ± 12.2	74.8 ± 22.4	0.460
UA (μmoI/L)	374.6 ± 37.0	373.7 ± 45.1	0.633	372.5 ± 31.9	371.3 ± 38.8	0.775	375.2 ± 38.2	373.9 ± 45.6	0.571
Na (mmol/L)	139.2 ± 4.05	139.2 ± 3.93	0.936	139.0 ± 4.0	139.3 ± 3.7	0.487	139.2 ± 4.1	139.2 ± 3.9	0.730
K (mmol/L)	4.02 ± 0.31	4.02 ± 0.27	0.974	4.05 ± 0.35	4.00 ± 0.26	0.201	4.02 ± 0.31	4.02 ± 0.27	0.570
TP (g/L)	70.2 ± 3.8	70.4 ± 3.9	0.150	70.2 ± 3.4	69.9 ± 3.8	0.471	70.2 ± 4.0	70.5 ± 3.9	0.109
Alb (g/L)	42.1 ± 3.2	42.3 ± 3.2	0.339	42.3 ± 3.5	42.3 ± 3.2	0.907	42.1 ± 3.1	42.3 ± 3.2	0.248
CPVA, *n* (%)	682 (100)	2005 (100)	1	140 (100)	187 (100)	1	542 (100)	1818 (100)	1
Roofline, *n* (%)	579 (84.9)	1719 (85.7)	0.591	119 (85.0)	156 (83.4)	0.700	460 (84.9)	1563 (86.0)	0.520
MI, *n* (%)	519 (76.1)	1499 (74.8)	0.486	105 (75.0)	136 (72.7)	0.644	414 (76.4)	1363 (75.0)	0.504
CTI, *n* (%)	622 (91.3)	1821 (90.8)	0.766	131 (93.6)	169 (90.4)	0.091	491 (90.6)	1652 (90.9)	0.844
CFAE, *n* (%)	298 (43.7)	849 (42.3)	0.538	57 (40.7)	90 (48.1)	0.182	241 (44.5)	759 (41.7)	0.261
SVC, *n* (%)	163 (23.9)	481 (24.0)	0.962	21 (15.0)	52 (27.8)	0.006	142 (26.2)	429 (23.6)	0.214
others, *n* (%)	91 (13.3)	288 (14.4)	0.508	18 (12.9)	30 (16.0)	0.421	73 (13.5)	258 (14.2)	0.671

Abbreviations: AF, atrial fibrilltion; Alb, albumin; ALT, alanine transaminase; AST, aspartate transaminase; BMI, body mass index; CAD, coronary artery disease; CFAE, complex fractionated atrial electrograms; Cr, creatinine; CPVA, circumferential pulmonary vein ablation; CTI, cavotricuspid isthmus; DBP, diastolic blood pressure; DM, diabetes mellitus; FBG, fast blood glucose; HDL, high‐density lipoprotein cholesterol; HGB, hemoglobin; HF, heart failure; HR, heart rate; HT, hypertension; LA, left atrium; LDL, low‐density lipoprotein cholesterol; LVEF, left ventricular ejection fraction; MI, mitral isthmus; PLT, platelet; RBC, red blood cell; SBP, systolic blood pressure; SVC, Superior vena cava; TC, total cholesterol; TG, triglyceride; TP, total protein; UA, uric acid; WBC, white blood cell.

### 3.2 Baseline characteristics between the two groups

According to the presence of VC, PsAF patients were classified into two groups: the calcification group (*n* = 327) and the no calcification group (*n* = 2360). There were 239 male patients in the calcification group and 1679 male patients in the no calcification group (*p* = 0.466). The incidence of DM, alcohol consumption, and AF recurrence were significantly higher in the calcification group than in the no calcification group (all *p* < 0.05). Similarly, the calcification group had significantly higher FBG, TG, TC, and LDL‐C levels than the control group (all *p* < 0.05). However, no significant differences were detected in the ablation methods between the two groups. In addition, there was a significant difference between the groups in terms of age, 63.0 ± 9.9 versus 60.7 ± 10.3 (*p* < 0.001, Table 2).

**Table 2:** Clinical characteristics between calification and no valvular calcification.

Variables	Calcification	No calcification	*χ* ^2^/*t*	*p* Value
	*n* = 327	*n* = 2360		
Male, *n* (%)	239 (73.1)	1679 (71.1)	0.532	0.466
Age (years)	63.0 ± 9.9	60.7 ± 10.3	−3.733	<0.001
BMI (Kg/m)^2^	25.6 ± 2.8	25.3 ± 2.8	−2.207	0.027
HT, *n* (%)	144 (44.0)	1063 (45.0)	0.117	0.732
Recurrence, *n* *(*%)	122 (37.3)	437 (18.5)	61.558	<0.001
Course of AF (month)	19.7 ± 5.9	19.1 ± 5.0	−1.489	0.137
Ascending aorta inner diameter (mm)	36.0 ± 2.9	35.8 ± 2.8	−0.839	0.402
LA (mm)	38.6 ± 5.3	37.8 ± 5.1	−2.834	0.005
HF, *n* (%)	21 (6.4)	134 (5.7)	0.293	0.589
CAD, *n* (%)	56 (17.1)	366 (15.5)	0.567	0.451
DM, *n* (%)	83 (25.4)	437 (18.5)	8.673	0.003
Stroke, *n* (%)	42 (12.8)	268 (11.4)	0.623	0.430
Alcohol consumption, *n* (%)	73 (22.3)	411 (17.4)	4.686	0.030
SBP (mmHg)	126.4 ± 16.0	126.6 ± 15.3	0.243	0.808
DBP (mmHg)	79.3 ± 11.8	80.6 ± 20.6	1.148	0.251
HR (bpm)	78.5 ± 13.3	79.1 ± 14.1	0.649	0.517
FBG (mmol/L)	6.65 ± 1.27	6.18 ± 1.05	−7.533	<0.001
TG (mmol/L)	1.59 ± 0.61	1.48 ± 0.45	−4.125	<0.001
TC (mmol/L)	4.03 ± 1.08	3.88 ± 1.15	−2.288	0.022
LDL‐C (mmol/L)	3.10 ± 1.12	2.95 ± 1.09	−2.229	0.026
HDL‐C (mmol/L)	1.62 ± 0.36	1.64 ± 0.37	0.776	0.438
WBC (10^12^/L)	8.34 ± 2.16	8.28 ± 2.02	−0.556	0.578
HGB (g/L)	141.4 ± 19.4	143.7 ± 17.9	2.221	0.026
PLT (10^9^/L)	173.5 ± 33.3	175.1 ± 33.7	0.815	0.415
RBC (10^12^/L)	4.15 ± 1.02	4.22 ± 1.14	1.031	0.303
Lymphocytes (10^12^/L)	2.04 ± 0.59	2.10 ± 0.59	1.713	0.087
Neutrophil (10^12^/L)	4.36 ± 1.54	4.38 ± 1.46	0.243	0.808
Monocytes (10^12^/L)	0.53 ± 0.15	0.51 ± 0.14	−1.701	0.089
ALT (μmol/L)	23.0 ± 5.4	23.2 ± 7.4	0.285	0.776
LVEF (%)	55.0 ± 4.1	54.9 ± 3.9	−0.388	0.698
AST (μmol/L)	15.2 ± 5.1	15.3 ± 5.9	0.359	0.720
Urea (mmol/L)	5.37 ± 1.39	5.34 ± 1.44	−0.450	0.653
Cr (μmoI/L)	74.5 ± 12.8	74.6 ± 20.5	0.129	0.898
UA (μmoI/L)	371.8 ± 36.6	374.2 ± 44.0	0.943	0.346
Na (mmol/L)	139.2 ± 3.83	139.2 ± 3.83	−0.222	0.824
K (mmol/L)	4.02 ± 0.30	4.02 ± 0.28	0.031	0.975
TP (g/L)	70.1 ± 3.6	70.4 ± 3.9	1.617	0.106
Alb (g/L)	42.3 ± 3.3	42.2 ± 3.2	−0.259	0.106
CPVA, *n* (%)	327 (100)	2360 (100)	‐	‐
Roofline, *n* (%)	275 (84.1)	2023 (85.7)	0.611	0.435
MI, *n* (%)	241 (73.7)	1777 (75.3)	0.391	0.532
CTI, *n* (%)	300 (91.7)	2143 (90.8)	0.306	0.580
CFAE, *n* (%)	147 (45.0)	1000 (42.4)	0.782	0.376
SVC, *n* (%)	73 (22.3)	571 (24.2)	0.552	0.458
others, *n* (%)	48 (14.7)	331 (14.0)	0.101	0.750

Abbreviations: AF, atrial fibrilltion; Alb, albumin; ALT, alanine transaminase; AST, aspartate transaminase; BMI, body mass index; CAD, coronary artery disease; CFAE, complex fractionated atrial electrograms; CPVA, circumferential pulmonary vein ablation; Cr, creatinine; CTI, cavotricuspid isthmus; DBP, diastolic blood pressure; DM, diabetes mellitus; FBG, fast blood glucose; HDL, high‐density lipoprotein cholesterol; HGB, hemoglobin; HF, heart failure; HR, heart rate; HT, hypertension; LA, left atrium; LDL, low‐density lipoprotein cholesterol; LVEF, left ventricular ejection fraction; MI, mitral isthmus; PLT, platelet; RBC, red blood cell; SBP, systolic blood pressure; SVC, superior vena cava; TC, total cholesterol; TG, triglyceride; TP, total protein; UA, uric acid; WBC, white blood cell.

### 3.3 VC number and clinical characteristics

Individuals were classified into four groups: 2360 (87.8%), 249 (9.3%), 49 (1.8%), and 29 (1.1%) who had VC of 0, 1, 2, and 3 by echocardiography, respectively (Figure S2). Recurrence was present in 437 (18.5%), 82 (34.9%), 24 (39.3%), and 13 (52.0%) of the four groups, which indicated statistically significant differences (*p* < 0.05, see Table 3). The prevalence of HF, CAD, DM, stroke, and alcohol consumption was not significantly different among the groups (all *p* > 0.05). BMI, FBG, TG, LDL‐C increased with increasing number. For comparison among groups, the difference was the most in FBG (all *p* < 0.001, Table 3).

**Table 3:** Baseline characteristics of the number of valvular calcification.

Variables	The number of the presence of valvular calcification					
	0	1	2	3	*p* Value	*p*＜0.05
	*n* = 2360	*n* = 249	*n* = 49	*n* = 29		
Male, *n* (%)	1679 (71.1)	184 (73.9)	36 (73.5)	19 (65.5)	0.699	
Age (years)	60.7 ± 10.3	62.1 ± 10.1	65.3 ± 7.9	66.3 ± 10.3	<0.001	a, b, c, e
BMI (Kg/m)^2^	25.3 ± 2.8	25.4 ± 3.0	25.7 ± 1.7	27.2 ± 2.3	0.002	c, e, f
Recurrence, *n* (%)	437 (18.5)	82 (34.9)	24 (39.3)	13 (52.0)	<0.001	a, b, c, e, f
HT, *n* (%)	1063 (45.0)	111 (44.6)	24 (49.0)	9 (31.0)	0.455	
Course of AF (month)	19.1 ± 5.0	20.1 ± 5.9	18.7 ± 5.3	19.2 ± 6.3	0.078	a
Ascending aorta inner diameter (mm)	35.8 ± 2.8	35.9 ± 2.9	36.2 ± 3.1	36.0 ± 2.8	0.724	
LA (mm)	37.8 ± 5.1	38.6 ± 5.3	38.3 ± 4.9	39.6 ± 6.3	0.024	a, c
HF, *n* (%)	134 (5.7)	16 (6.4)	3 (6.1)	2 (6.9)	0.958	
CAD, *n* (%)	366 (15.5)	45 (18.1)	7 (14.3)	4 (13.8)	0.734	
DM, *n* (%)	437 (18.5)	56 (22.5)	6 (12.2)	6 (20.7)	0.467	b, c, d, e
Stroke, *n* (%)	268 (11.4)	30 (12.0)	7 (11.5)	5 (20.0)	0.711	
Alcohol consumption, *n* (%)	411 (17.4)	55 (22.1)	11 (22.4)	7 (24.1)	0.190	
SBP (mmHg)	126.6 ± 15.3	126.9 ± 16.1	126.9 ± 15.3	121.5 ± 15.4	0.352	
DBP (mmHg)	80.6 ± 20.6	79.5 ± 11.9	79.9 ± 10.9	76.6 ± 12.3	0.589	
HR (bpm)	79.1 ± 14.1	78.5 ± 12.8	80.0 ± 15.9	77.4 ± 12.6	0.637	
FBG (mmol/L)	6.19 ± 1.06	6.60 ± 1.25	6.70 ± 1.11	7.28 ± 1.57	<0.001	a, b, c, e
TG (mmol/L)	1.48 ± 0.45	1.56 ± 0.67	1.65 ± 0.36	1.75 ± 0.48	<0.001	a, b, c, e
TC (mmol/L)	3.88 ± 1.15	3.99 ± 1.08	4.14 ± 0.86	4.18 ± 1.33	0.092	
LDL‐C (mmol/L)	2.95 ± 1.09	3.05 ± 1.13	3.07 ± 1.01	3.52 ± 1.06	0.020	c, e
HDL‐C (mmol/L)	1.64 ± 0.37	1.64 ± 0.38	1.59 ± 0.27	1.55 ± 0.28	0.487	
WBC (10^12^/L)	8.28 ± 2.02	8.36 ± 2.12	8.52 ± 2.26	7.94 ± 2.36	0.623	
HGB (g/L)	143.7 ± 17.9	141.8 ± 19.5	139.9 ± 18.8	141.7 ± 19.1	0.112	
PLT (10^9^/L)	175.1 ± 33.7	174.6 ± 33.0	167.2 ± 30.8	178.8 ± 35.0	0.165	b
RBC (10^12^/L)	4.22 ± 1.14	4.15 ± 1.04	4.29 ± 0.87	3.97 ± 1.05	0.459	
Lymphocytes (10^12^/L)	2.10 ± 0.59	2.08 ± 0.56	2.00 ± 0.71	1.78 ± 0.55	0.018	c, e
Neutrophil (10^12^/L)	4.38 ± 1.46	4.34 ± 1.51	4.69 ± 1.70	4.03 ± 1.40	0.262	
Monocytes (10^12^/L)	0.51 ± 0.14	0.52 ± 0.14	0.56 ± 0.20	0.49 ± 0.12	0.075	b
ALT (μmol/L)	23.2 ± 7.4	23.2 ± 5.6	23.4 ± 4.5	21.4 ± 5.0	0.606	
LVEF (%)	54.9 ± 3.9	54.9 ± 4.1	55.8 ± 4.7	54.9 ± 3.4	0.424	
AST (μmol/L)	15.3 ± 5.9	15.5 ± 5.5	14.4 ± 3.0	14.0 ± 3.5	0.368	
Urea (mmol/L)	5.34 ± 1.44	5.38 ± 1.43	5.35 ± 1.07	5.31 ± 1.28	0.963	
Cr (μmoI/L)	74.6 ± 20.5	74.3 ± 13.1	77.2 ± 10.6	71.4 ± 12.7	0.645	
UA (μmoI/L)	374.2 ± 44.0	370.5 ± 39.1	378.3 ± 24.8	372.0 ± 30.5	0.527	
Na (mmol/L)	139.2 ± 3.98	139.3 ± 3.81	138.3 ± 4.30	139.6 ± 2.96	0.384	
K (mmol/L)	4.02 ± 0.28	4.03 ± 0.32	3.99 ± 0.20	4.02 ± 0.22	0.836	
TP (g/L)	70.4 ± 3.9	70.0 ± 3.7	69.7 ± 3.4	70.5 ± 3.7	0.345	
Alb (g/L)	42.2 ± 3.2	42.3 ± 3.3	42.2 ± 3.1	41.8 ± 3.6	0.824	
CPVA, *n* (%)	2360 (100)	249 (100)	49 (100)	29 (100)	1	
Roofline, *n* (%)	2023 (85.7)	211 (84.7)	41 (83.7)	23 (79.3)	0.744	
MI, *n* (%)	1777 (75.3)	188 (75.5)	36 (73.5)	17 (58.6)	0.226	
CTI, *n* (%)	2143 (90.8)	230 (92.4)	45 (91.8)	25 (86.2)	0.682	
CFAE, *n* (%)	1000 (42.4)	115 (46.2)	23 (46.9)	9 (31.0)	0.346	
SVC, *n* (%)	571 (24.2)	59 (23.7)	8 (16.3)	6 (20.7)	0.611	
Others, *n* (%)	331 (14.0)	35 (14.1)	9 (18.4)	3 (13.8)	0.861	

*Note*: a: 0 versus 1, b: 0 versus 2, c: 0 versus 3, d: 1 versus 2, e: 1 versus 3, f: 2 versus 3.

Abbreviations: AF, atrial fibrilltion; Alb, albumin; ALT, alanine transaminase; AST, aspartate transaminase; BMI, body mass index; CAD, coronary artery disease; CFAE, complex fractionated atrial electrograms; CPVA, circumferential pulmonary vein ablation; Cr, creatinine; CTI, cavotricuspid isthmus; DBP, diastolic blood pressure; DM, diabetes mellitus; FBG, fast blood glucose; HDL, high‐density lipoprotein cholesterol; HGB, hemoglobin; HF, heart failure; HR, heart rate; HT, hypertension; LA, left atrium; LDL, low‐density lipoprotein cholesterol; LVEF, left ventricular ejection fraction; MI, mitral isthmus; PLT, platelet; RBC, red blood cell; SBP, systolic blood pressure; SVC, superior vena cava; TC, total cholesterol; TG, triglyceride; TP, total protein; UA, uric acid; WBC, white blood cell.

### 3.4 VC and recurrence

Table 4 shows the results of multivariate logistic regression for the association between the incidence of recurrence and VC. There were seven models after adjusting for age, sex, ascending aorta inner diameter, LA, LVEF, course of AF, BMI, HT, HF, CAD, DM, stroke, alcohol consumption, SBP, DBP, HR, WBC, RBC, PLT, HGB, lymphocytes, neutrophil, monocytes, ALT, AST, TG, TC, HDL, LDL, urea, Cr, UA, FBG, Na, K, TP, Alb, CPVA, Roofline, MI, CFAE, SVC, and others. The ORs were 2.419 (1.889−3.097), 2.533 (1.937−3.314), 2.455 (1.868−3.226), 2.466 (1.874−3.245), 2.371 (1.797−3.128), 2.293 (1.732–3.036), and 2.286 (1.726−3.027) for VC in models 1, 2, 3, 4, 5, 6, and 7, respectively (all *p* < 0.05) (Table 4).

**Table 4:** Odds ratio and 95% confidence interval for recurrence.

Models	Valvular calcification	
	OR (95% CI)	*p* Value
Model 1	2.419 (1.889−3.097)	<0.001
Model 2	2.533 (1.937−3.314)	<0.001
Model 3	2.455 (1.868−3.226)	<0.001
Model 4	2.466 (1.874−3.245)	<0.001
Model 5	2.371 (1.797−3.128)	<0.001
Model 6	2.293 (1.732−3.036	<0.001
Model 7	2.286 (1.726−3.027)	<0.001

Abbreviations: AF, atrial fibrilltion; Alb, albumin; ALT, alanine transaminase; AST, aspartate transaminase; BMI, body mass index; CAD, coronary artery disease; CFAE, complex fractionated atrial electrograms; CPVA, circumferential pulmonary vein ablation; Cr, creatinine; CTI, cavotricuspid isthmus; DBP, diastolic blood pressure; DM, diabetes mellitus; FBG, fast blood glucose; HDL, high‐density lipoprotein cholesterol; HGB, hemoglobin; HF, heart failure; HR, heart rate; HT, hypertension; LA, left atrium; LDL, low‐density lipoprotein cholesterol; LVEF, left ventricular ejection fraction; MI, mitral isthmus; PLT, platelet; RBC, red blood cell; SBP, systolic blood pressure; SVC, superior vena cava; TC, total cholesterol; TG, triglyceride; TP, total protein; UA, uric acid; WBC, white blood cell.

Model 1: adjusted for age, sex, ascending aorta inner diameter, LA, and LVEF.

Model 2: adjusted for age, sex, ascending aorta inner diameter, LA, LVEF, course of AF, BMI, HT, and HF.

Model 3: adjusted for age, sex, ascending aorta inner diameter, LA, LVEF, course of AF, BMI, HT, HF, CAD, DM, stroke, alcohol consumption, SBP, and DBP.

Model 4: adjusted for age, sex, ascending aorta inner diameter, LA, LVEF, course of AF, BMI, HT, HF, CAD, DM, stroke, alcohol consumption, SBP, DBP, HR, WBC, RBC, PLT, HGB, lymphocytes, neutrophil, and monocytes.

Model 5: adjusted for age, sex, ascending aorta inner diameter, LA, LVEF, course of AF, BMI, HT, HF, CAD, DM, stroke, alcohol consumption, SBP, DBP, HR, WBC, RBC, PLT, HGB, lymphocytes, neutrophil, monocytes, ALT, AST, TG, TC, HDL, and LDL.

Model 6: adjusted for age, sex, ascending aorta inner diameter, LA, LVEF, course of AF, BMI, HT, HF, CAD, DM, stroke, alcohol consumption, SBP, DBP, HR, WBC, RBC, PLT, HGB, lymphocytes, neutrophil, monocytes, ALT, AST, TG, TC, HDL, LDL, urea, Cr, UA, FBG, Na, K, TP, and Alb.

Model 7: adjusted for age, sex, ascending aorta inner diameter, LA, LVEF, course of AF, BMI, HT, HF, CAD, DM, stroke, alcohol consumption, SBP, DBP, HR, WBC, RBC, PLT, HGB, lymphocytes, neutrophil, monocytes, ALT, AST, TG, TC, HDL, LDL, Urea, Cr, UA, FBG, Na, K, TP, Alb, CPVA, Roofline, MI, CFAE, SVC, and others.

### 3.5 Score system of AF recurrence and ROC

Multivariate logistic regression analyses were used to explore the association of risk factors with AF recurrence. There were seven independent risk factors for AF recurrence: VC, LA, course of AF, BMI, TC, FBG, and alcohol consumption. ROC curves were generated and sensitivity and specificity were calculated. The cut‐off value of LA, course of AF, BMI, TC, and FBG was obtained by the maximum Jordan index. According to the cut‐off value, the continuous variables were divided into the categorical variables. Based on the regression coefficient, points were assigned to those factors and subsequently summed to obtain a predictive score of AF recurrence. The points were as follows: VC—3, LA > 37 mm—3, course of AF > 19 months—2, BMI > 26.99—2, TC > 3.65 mmol/L—1, FBG > 7.10 mmol/L—1, and alcohol consumption—2 (Table 5). Overall, 133 patients were included between February 2022 and May 2022. ROC analyses were used to evaluate the discrimination of the model; the AUC was 0.787 (95% confidence interval [CI]: 0.700−0.873). The optimal cutoff value was 7. In addition, the sensitivity was 70.3%, and the specificity was 71.9% (Figure S3).

**Table 5:** The logistic multivariable logistic regression analyses of AF recurrence.

Variables	OR (95% CI)	*p* Value	Regression coefficeint	Point
Valvular calcification	2.291 (1.843−2.703)	<0.001	0.829	3
LA > 37 mm	2.325 (1.907−2.834)	<0.001	0.844	3
Course of AF > 19 months	2.231 (1.842−2.703)	<0.001	0.803	2
BMI > 26.99	1.648 (1.331−2.040)	<0.001	0.500	2
TC > 3.65 mmol/L	1.477 (1.211−1.801)	<0.001	0.390	1
FBG > 7.10 mmol/L	1.358 (1.075−1.716)	<0.001	0.306	1
Alcohol consumption	1.875 (1.488−2.362)	<0.001	0.629	2

Abbreviations: AF, atrial fibrilltion; BMI, body mass index; FBG, fast blood glucose; LA, left atrium; TC, total cholesterol.

## 4 DISCUSSION

In this prospective study, we considered VC as a whole and explored the association of VC with the recurrence of PsAF. In addition, we deduced the scoring system for diagnosis and risk assessment in the clinical setting. Moreover, we demonstrated an increased risk of PsAF recurrence with an increase in the VC number. In addition, the scoring system exhibited excellent prediction ability regarding PsAF recurrence, which contributes to interventional strategies and individualizing treatments for doctors and is expected to provide new insights concerning prognosis.

As a progressive disease, VC is characterized by ectopic calcification in the aortic valvular, mitral valve, and tricuspid valve, which is also associated with the incidence of cardiovascular diseases such as HF and CAD.^14^ The pathogenic mechanism for VC is not yet fully understood. Altered shear forces are initiated in valvular tissue, which might cause an imbalance between oxidative stress and antioxidants.^15^ In addition, the dysfunction of endothelial structure precedes calcific mineral deposition with a series of inflammatory reactions.^16^ Besides, apoptosis also plays a critical role in the VC process, which releases calcium and forms calcified nodules.^17^ For the aortic valve, there are two cell types in valve leaflets, valvular endothelial cells and valvular interstitial cells, which are influenced by blood flow and shear stress.^18^ With stronger shear stress, valvular endothelial cells trigger a series of signaling pathways, such as oxidative stress, inflammatory infiltration, and lipid deposition.^19^

In clinical practice, VC is asymptomatic and usually discovered incidentally in physical examination. Recent studies have proven the association between VC and cardiovascular diseases, especially arrhythmia. As early as 1994, mitral annular calcification (MAC) was linearly correlated with a higher prevalence of atrioventricular block and bundle branch block.^20^ A total of 342 participants who had undergone transcatheter aortic valve implantation were stratified into two groups according to whether atrioventricular block occurred. After multivariate analysis, AVC was observed to be an independent risk factor for atrioventricular block.^21^

Regarding AF, numerous studies have found that MCV was associated with the incidence of AF. In the Multi‐Ethnic Study of Atherosclerosis, 5683 patients were classified into the MAC progression group and the no MAC progression group. After a median of 8.6 years of follow‐up, MAC progression was associated with an increased risk for AF.^22^ A meta‐analysis confirmed the prognostic value of MAC for predicting AF. In addition, AF patients with MAC had a higher risk of adverse events.^23^ It is speculated that this may be related to left atrial enlargement. The underlying mechanism is likely related to the sharing of numerous biological characteristics. However, most VC studies have focused on MAC, and there have been few studies on AVC and tricuspid valve calcification (TVC). As a type of AF, valvular AF has been gradually emphasized. The underlying mechanisms of VC formation also have many commonalities, such as oxidative stress and lipid deposits. Thus, we speculated that AVC and TVC may also be associated with AF.

In our study, VC was an independent risk factor for PsAF recurrence after RFCA. In addition, with the increase in VC number, the PsAF recurrence rate was higher. The underlying mechanisms by which VC increases PsAF recurrence are poorly understood and are regulated by multiple factors. First, VC could induce myocardial fibrosis and atrial remodeling, which also contribute to the outcome of AF after RFCA. Next, the pathophysiological mechanisms of VC are oxidative stress and endothelial dysfunction that trigger a series of downstream signaling pathways, which also influence the AF electroanatomical substrate. In addition, dyslipidemia, endothelial dysfunction, and insulin resistance are characteristics of VC, which synergize and are independent. These multiple factors contribute to AF progression. Yao et al. enrolled 785 AF patients who underwent ablation. After multivariate analysis, MAC was an independent risk factor for AF recurrence during the 16‐month follow‐up (1.48, 1.13−195).^24^ In our study, we explored VC integrally for the first time. With the increase in VC, the incidence of AF recurrence increased. We speculated that the burden of the dysfunction state was higher in the three valve calcifications.

There are several means of detecting VC, such as echocardiography, coronary computed tomography angiography (CCTA), and cardiac magnetic resonance imaging. For CCTA, VC was defined as a cutoff of 130 Hounsfield units of valve by the Agatston method.^25^ A recent study found that the quantitative analysis of VC might provide more information associated with death or valve replacement.^26^ As an invasive method, it is not used widely in clinical practice. Echocardiography not only accurately assesses cardiac structure but also provides semiquantitative VC. Global cardiac calcium scores and echocardiographic calcium scores were assessed by echocardiography. After analysis, higher calcium scores were significantly associated with the incidence of AF.^27^ In the Framingham study, MAC was also assessed by echocardiography.^7^ As an inexpensive and radiation‐free tool, echocardiography can precisely present calcification of valve leaves. Therefore, we chose echocardiography, which may be more suitable in our study.

There are many factors which could influence VC, such as aged, fibrotic underlying processes. In elderly patients with cardiovascular disease, the prevalence of MAC was 42%.^28^ As known the “AF begets AF” hypothesis, frequent AF attacks might lead to the remodeling of structural and electrical and the perpetuation AF. Due to the similar of the underlying mechanisms between AF and CV, this may be the “CV begets AF.” In our model, VC, LAD, course of AF, BMI, TC, FBG, and alcohol consumption were the independent risk factors for AF recurrence, which indicated that the model had the excellent predictive value. As the classical factors, LAD, course of AF, and alcohol consumption reflect the the extent of atrial fibrosis, which was confirmed in numerous studies.^29^, ^30^, ^31^ A meta analysis enrolled the 26 studies which found that BMI was associated with the AF recurrence.^32^ A recent study found that the level of TC and FBG were higher in recurrence group.^33^ For the model, those factors might reflect the state of inflammatory, atrial fibrosis, lipid metabolism in PsAF patients, which provided individualized assessments in clinical practice.

## 5 LIMITATIONS

Our study has some limitations. First, AF recurrence is complex and multifactorial. The single‐center nature of this study and the relatively small number of enrolled patients may have introduced selection bias. Second, VC was qualitatively evaluated in our study. Quantitative assessment will be performed for further justification. Furthermore, pulmonary valve calcification was not assessed in our study. What remains to be determined is whether the two are connected.

## 6 FUTURE DIRECTIONS

Our study suggested that VC can predict the PsAF recurrence. However, future studies are needed to explore relationship between the severity of VC and AF recurrence. Besides, the the mechanism of VC in the pathogenesis of AF will decipher.

## 7 CONCLUSIONS

In PsAF, VC was an independent risk factor for recurrence after RFCA. With the increase in VC number, the incidence of recurrence was higher. The scoring system had better predictive value based on VC.

## CONFLICT OF INTEREST STATEMENT

The authors declare no conflict of interest.

## Supporting information


**Fig. S1** Population flowchart of enroll patients.

**Fig. S2** Valvular calcification of persistent atrial fibrillation recurrence. *:Vs 0 < 0.05；#:Vs 1 < 0.05；&:Vs 2 < 0.05.

**Fig. S3** ROC curve analysis of the score system for the prediction of recurrence.

## Data Availability

Data is available on request from the authors.

## References

[1] Kim J , Kim JY , Jeong DS , et al. Long‐term outcome of thoracoscopic ablation and radiofrequency catheter ablation for persistent atrial fibrillation as a de novo procedure. Europace. 2023;25(5):euad096. 37144277 10.1093/europace/euad096 PMC10228598

[2] Dong Z , Du X , Hou XX , He L , Dong JZ , Ma CS . Effect of electrical cardioversion on 1‐year outcomes in patients with early recurrence after catheter ablation for atrial fibrillation. Clin Cardiol. 2021;44(8):1128‐1138. 34101841 10.1002/clc.23663 PMC8364725

[3] Bergonti M , Spera FR , Ferrero TG , et al. Anterior mitral line in patients with persistent atrial fibrillation and anterior scar: a multicenter matched comparison‐The MiLine study. Heart Rhythm. 2023;20(5):658‐665. 36640853 10.1016/j.hrthm.2023.01.009

[4] Obisesan OH , Kou M , Wang FM , et al. Lipoprotein(a) and subclinical vascular and valvular calcification on cardiac computed tomography: the atherosclerosis risk in communities study. J Am Heart Assoc. 2022;11(11):e024870. 35656990 10.1161/JAHA.121.024870 PMC9238743

[5] Fashanu OE , Upadhrasta S , Zhao D , et al. Effect of progression of valvular calcification on left ventricular structure and frequency of incident heart failure (from the Multiethnic Study of Atherosclerosis). Am J Cardiol. 2020;134:99‐107. 32917344 10.1016/j.amjcard.2020.08.017 PMC7572881

[6] Wada S , Iwanaga Y , Nakai M , Miyamoto Y , Noguchi T . Aortic valve and aortic root calcifications for predicting major adverse cardiovascular events: NADESICO study. Heart Vessels. 2023;38(4):562‐569. 36224476 10.1007/s00380-022-02187-9

[7] Fox CS , Parise H , Vasan RS , et al. Mitral annular calcification is a predictor for incident atrial fibrillation. Atherosclerosis. 2004;173(2):291‐294. 15064104 10.1016/j.atherosclerosis.2003.12.018

[8] Andrade JG , Aguilar M , Atzema C , et al. The 2020 Canadian Cardiovascular Society/Canadian Heart Rhythm Society Comprehensive Guidelines for the management of atrial fibrillation. Can J Cardiol. 2020;36(12):1847‐1948. 33191198 10.1016/j.cjca.2020.09.001

[9] Cheng Y , Lu Z , Cao X , Ding X , Zou J , Jin H . Predictive role of cardiac valvular calcification in all‐cause mortality of Chinese initial haemodialysis patients: a follow‐up study of 4 years. BMC Nephrol. 2023;24(1):37. 36792978 10.1186/s12882-023-03076-7 PMC9933363

[10] Writing Committee Members , Lawton JS , Tamis‐Holland JE , Bangalore S , et al. 2021 ACC/AHA/SCAI guideline for coronary artery revascularization. JACC. 2022;79(2):e21‐e129. 34895950 10.1016/j.jacc.2021.09.006

[11] Ducharme A , Zieroth S , Ahooja V , et al Canadian Cardiovascular Society‐Canadian Heart Failure Society focused clinical practice update of patients with differing heart failure phenotypes. Can J Cardiol. 2023;39(8):1030‐1040. 37169222 10.1016/j.cjca.2023.04.022

[12] Heran M , Lindsay P , Gubitz G , et al. Canadian stroke best practice recommendations: acute stroke management, 7th edition practice guidelines update, 2022. *Canadian J Neurol Sci/J Canadien des Sciences Neurologiques*. Published online December 19, 2022:1‐31. 10.1017/cjn.2022.344 36529857

[13] ElSayed NA , Aleppo G , Aroda VR , et al. Summary of revisions: standards of care in diabetes‐2023. Diabetes Care. 2023;46(suppl 1):S5‐S9. 36507641 10.2337/dc23-Srev PMC9810459

[14] Bäck M , Michel JB . From organic and inorganic phosphates to valvular and vascular calcifications. Cardiovasc Res. 2021;117(9):2016‐2029. 33576771 10.1093/cvr/cvab038 PMC8318101

[15] Shu L , Yuan Z , Li F , Cai Z . Oxidative stress and valvular endothelial cells in aortic valve calcification. Biomed Pharmacother. 2023;163:114775. 37116353 10.1016/j.biopha.2023.114775

[16] Clemente A , Traghella I , Mazzone A , et al. Vascular and valvular calcification biomarkers. Adv Clin Chem. 2020;95:73‐103. 32122525 10.1016/bs.acc.2019.08.002

[17] Rodriguez KJ , Piechura LM , Porras AM , Masters KS . Manipulation of valve composition to elucidate the role of collagen in aortic valve calcification. BMC Cardiovasc Disord. 2014;14:29. 24581344 10.1186/1471-2261-14-29 PMC3946110

[18] Blaser MC , Kraler S , Lüscher TF , Aikawa E . Multi‐omics approaches to define calcific aortic valve disease pathogenesis. Circ Res. 2021;128(9):1371‐1397. 33914608 10.1161/CIRCRESAHA.120.317979 PMC8095729

[19] Carbone RG . Advancements in calcific aortic valve disease. Int J Cardiol. 2022;358:85‐86. 35469939 10.1016/j.ijcard.2022.04.052

[20] Abramowitz Y , Jilaihawi H , Chakravarty T , Mack MJ , Makkar RR . Mitral annulus calcification. JACC. 2015;66(17):1934‐1941. 26493666 10.1016/j.jacc.2015.08.872

[21] Pollari F , Großmann I , Vogt F , et al. Risk factors for atrioventricular block after transcatheter aortic valve implantation: a single‐centre analysis including assessment of aortic calcifications and follow‐up. EP Europace. 2019;21(5):787‐795. 30629159 10.1093/europace/euy316

[22] O'Neal WT , Efird JT , Nazarian S , et al. Mitral annular calcification progression and the risk of atrial fibrillation: results from MESA. Eur Heart J‐Cardiovasc Imag. 2018;19(3):279‐284. 10.1093/ehjci/jex093 PMC5837370 28460029

[23] Li Y , Lu Z , Li X , Huang J , Wu Q . Mitral annular calcification is associated with atrial fibrillation and major cardiac adverse events in atrial fibrillation patients: a systematic review and meta‐analysis. Medicine. 2019;98(44):e17548. 31689756 10.1097/MD.0000000000017548 PMC6946188

[24] Yao Y , Zhang Z , Xue J , et al. Echocardiographic mitral annular calcification is associated with atrial fibrillation recurrence after catheter ablation. Am J Cardiol. 2023;193:55‐60. 36871530 10.1016/j.amjcard.2023.01.054

[25] Williams MC , Massera D , Moss AJ , et al. Prevalence and clinical implications of valvular calcification on coronary computed tomography angiography. Eur Heart J‐Cardiovasc Imag. 2021;22(3):262‐270. 10.1093/ehjci/jeaa263 PMC7899264 33306104

[26] Pawade T , Clavel MA , Tribouilloy C , et al. Computed tomography aortic valve calcium scoring in patients with aortic stenosis. Circulation: Cardiovasc Imag. 2018;11(3):e007146. 10.1161/CIRCIMAGING.117.007146 29555836

[27] Li TYW , Yeo LLL , Ho JSY , et al. Association of global cardiac calcification with atrial fibrillation and recurrent stroke in patients with embolic stroke of undetermined source. J Am Soc Echocardiogr. 2021;34(10):1056‐1066. 33872703 10.1016/j.echo.2021.04.008

[28] Barasch E , Gottdiener JS , Larsen EK , Chaves PH , Newman AB , Manolio TA . Clinical significance of calcification of the fibrous skeleton of the heart and aortosclerosis in community dwelling elderly. Am Heart J. 2006;151(1):39‐47. 16368289 10.1016/j.ahj.2005.03.052

[29] Khan HR , Yakupoglu HY , Kralj‐Hans I , et al. Left atrial function predicts atrial arrhythmia recurrence following ablation of long‐standing persistent atrial fibrillation. Circulation: Cardiovasc Imag. 2023;16(6):e015352. 10.1161/CIRCIMAGING.123.015352 PMC10281195 37288553

[30] Li Z , Wang S , Hidru TH , et al. Long atrial fibrillation duration and early recurrence are reliable predictors of late recurrence after radiofrequency catheter ablation. Front Cardiovasc Med. 2022;9:864417. 35402564 10.3389/fcvm.2022.864417 PMC8990906

[31] Grindal AW , Sparrow RT , McIntyre WF , Conen D , Healey JS , Wong JA . Alcohol consumption and atrial arrhythmia recurrence after atrial fibrillation ablation: a systematic review and meta‐analysis. Can J Cardiol. 2023;39(3):266‐273. 36549481 10.1016/j.cjca.2022.12.010

[32] Liu F , Song T , Hu Q , et al Body mass index and atrial fibrillation recurrence post ablation: a systematic review and dose‐response meta‐analysis. Front Cardiovasc. 2022;9:999845. 10.3389/fcvm.2022.999845 PMC9932032 36818915

[33] Song YQ , Xu WJ , Lin CL , Wu QM . GPIIb/IIIa expression changes in atrial fibrillation post radiofrequency ablation. Eur Rev Med Pharmacol Sci. 2018;22(20):6959‐6964. 30402862 10.26355/eurrev_201810_16166

