# Supplementary figures and images for: Predictive value of valvular calcification for the recurrence of persistent atrial fibrillation after radiofrequency catheter ablation
Source: Clin Cardiol. 2023 Nov 7;47(2):e24176. doi: 10.1002/clc.24176 (PMC10826787; doi:10.1002/clc.24176)

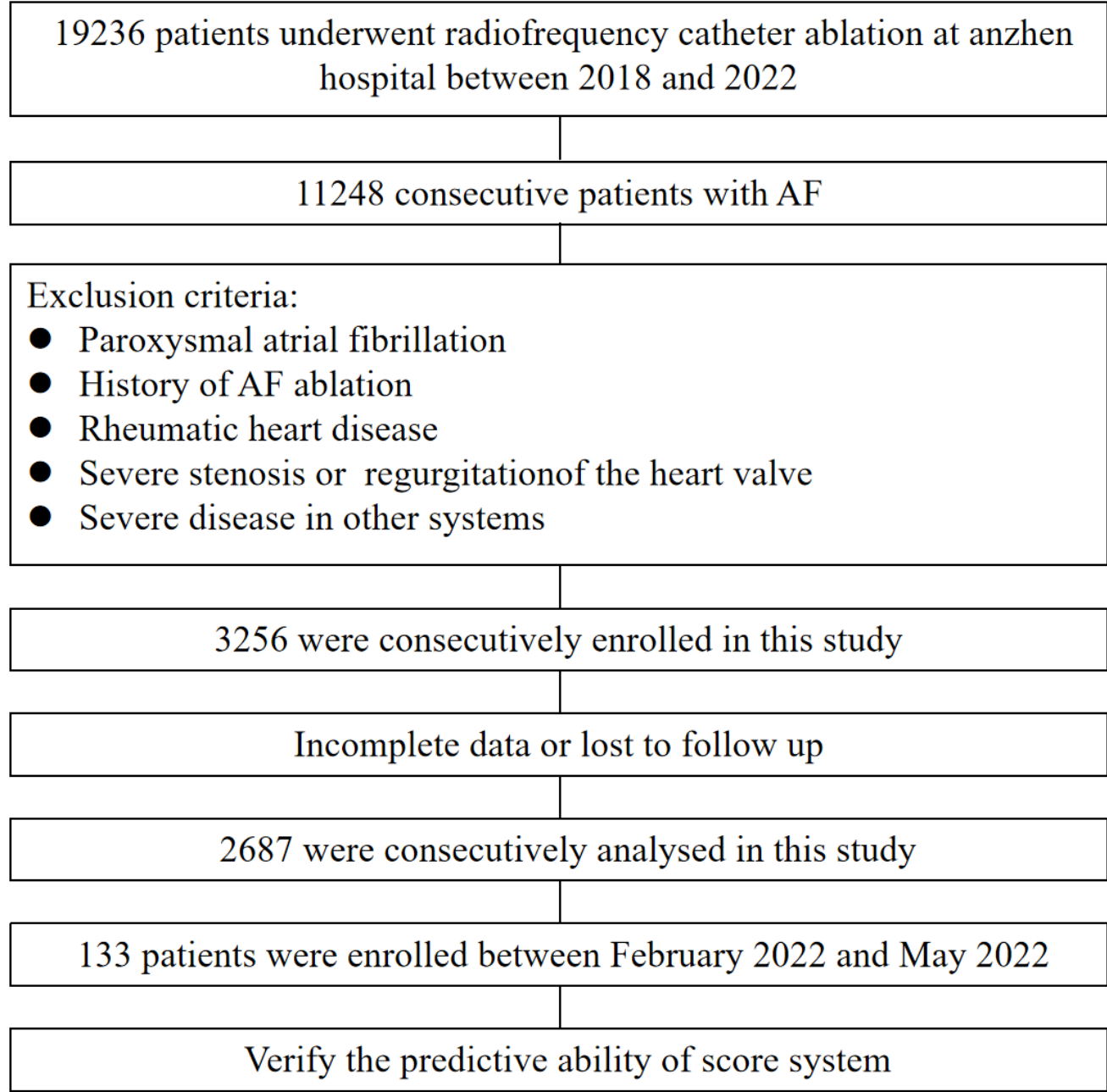

Supplement: Supplementary file 1 — Fig. S1 Population flowchart of enroll patients. [file CLC-47-e24176-s002.pdf]

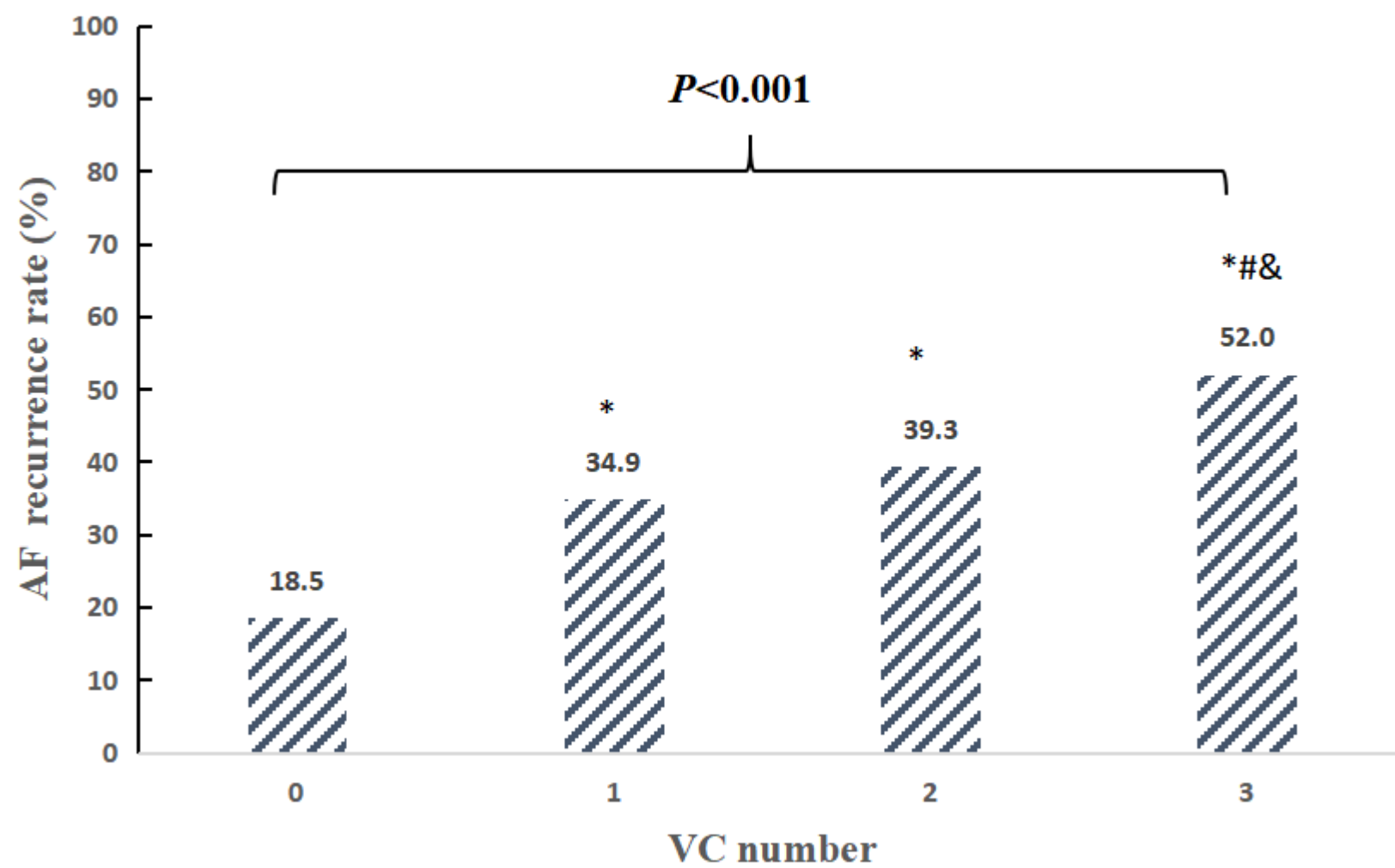

Supplement: Supplementary file 2 — Fig. S2 Valvular calcification of persistent atrial fibrillation recurrence. *:Vs 0 < 0.05；#:Vs 1 < 0.05；&:Vs 2 < 0.05. [file CLC-47-e24176-s001.pdf]

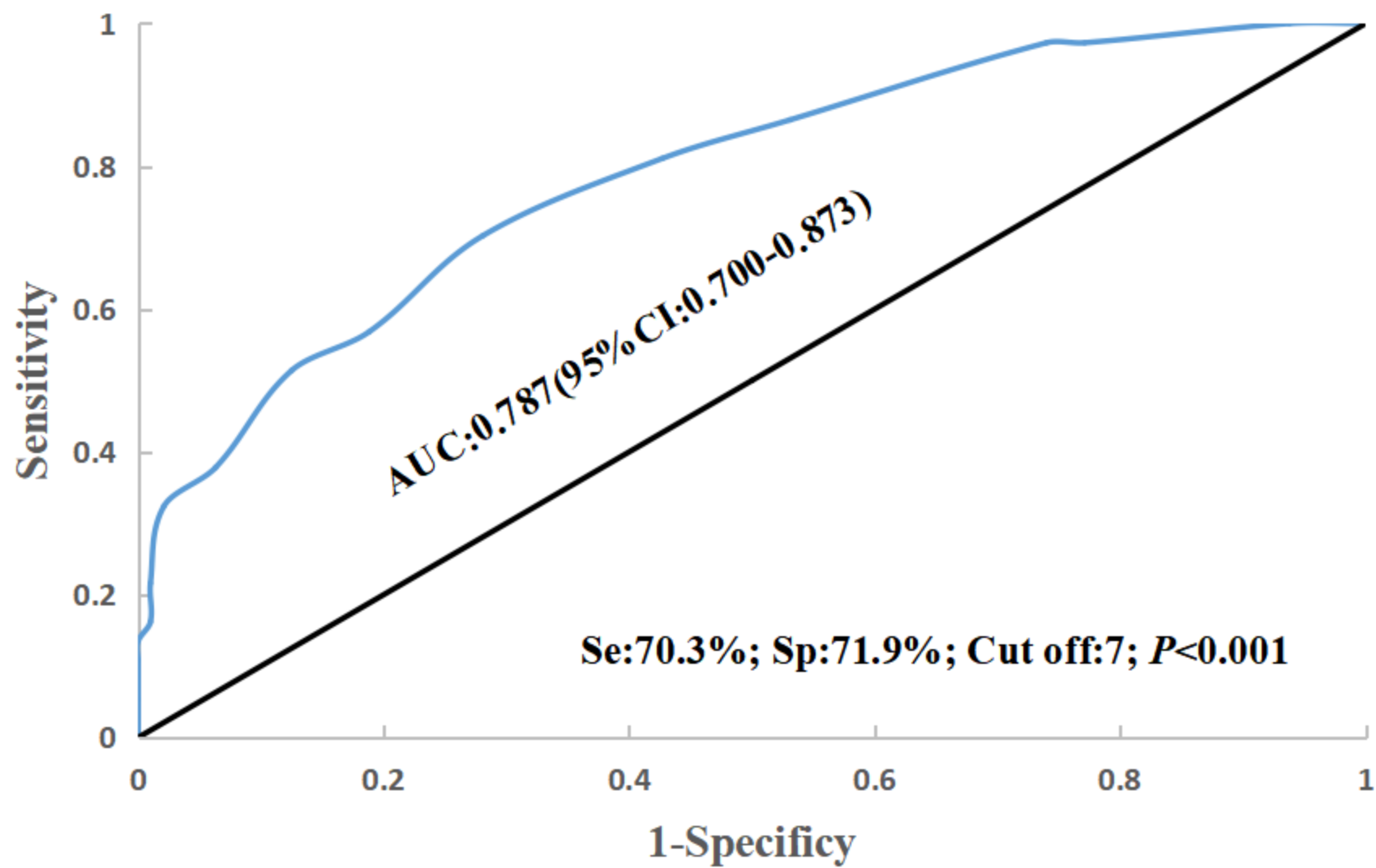

Supplement: Supplementary file 3 — Fig. S3 ROC curve analysis of the score system for the prediction of recurrence. [file CLC-47-e24176-s003.pdf]
